# Development and validation of a deep learning-based automatic segmentation model for assessing intracranial volume: comparison with NeuroQuant, FreeSurfer, and SynthSeg

**DOI:** 10.3389/fneur.2023.1221892

**Published:** 2023-09-01

**Authors:** Pae Sun Suh, Wooseok Jung, Chong Hyun Suh, Jinyoung Kim, Jio Oh, Hwon Heo, Woo Hyun Shim, Jae-Sung Lim, Jae-Hong Lee, Ho Sung Kim, Sang Joon Kim

**Affiliations:** ^1^Department of Radiology and Research Institute of Radiology, Asan Medical Center, University of Ulsan College of Medicine, Seoul, Republic of Korea; ^2^R&D Center, VUNO, Seoul, Republic of Korea; ^3^Department of Neurology, Asan Medical Center, University of Ulsan College of Medicine, Seoul, Republic of Korea

**Keywords:** deep learning, artificial intelligence, brain, intracranial volume segmentation, neurodegenerative disease

## Abstract

**Background and purpose:**

To develop and validate a deep learning-based automatic segmentation model for assessing intracranial volume (ICV) and to compare the accuracy determined by NeuroQuant (NQ), FreeSurfer (FS), and SynthSeg.

**Materials and methods:**

This retrospective study included 60 subjects [30 Alzheimer’s disease (AD), 21 mild cognitive impairment (MCI), 9 cognitively normal (CN)] from a single tertiary hospital for the training and validation group (50:10). The test group included 40 subjects (20 AD, 10 MCI, 10 CN) from the ADNI dataset. We propose a robust ICV segmentation model based on the foundational 2D UNet architecture trained with four types of input images (both single and multimodality using scaled or unscaled T1-weighted and T2-FLAIR MR images). To compare with our model, NQ, FS, and SynthSeg were also utilized in the test group. We evaluated the model performance by measuring the Dice similarity coefficient (DSC) and average volume difference.

**Results:**

The single-modality model trained with scaled T1-weighted images showed excellent performance with a DSC of 0.989 ± 0.002 and an average volume difference of 0.46% ± 0.38%. Our multimodality model trained with both unscaled T1-weighted and T2-FLAIR images showed similar performance with a DSC of 0.988 ± 0.002 and an average volume difference of 0.47% ± 0.35%. The overall average volume difference with our model showed relatively higher accuracy than NQ (2.15% ± 1.72%), FS (3.69% ± 2.93%), and SynthSeg (1.88% ± 1.18%). Furthermore, our model outperformed the three others in each subgroup of patients with AD, MCI, and CN subjects.

**Conclusion:**

Our deep learning-based automatic ICV segmentation model showed excellent performance for the automatic evaluation of ICV.

## Highlights

- The single-modality model trained with scaled T1-weighted images showed excellent performance with a DSC of 0.989 ± 0.002 and an average volume difference of 0.46 ± 0.38%.

- Our multimodality model trained with both unscaled T1-weighted and T2-FLAIR images showed similar performance with a DSC of 0.988 ± 0.002 and an average volume difference of 0.47% ± 0.35%.

- The overall average volume difference with our model showed relatively higher accuracy than NQ (2.15 ± 1.72%), FS (3.69 ± 2.93%), and SynthSeg (1.88 ± 1.18%).

## Introduction

Neurodegenerative disorders cause dementia and Alzheimer’s disease (AD) is the most common cause. AD initially presents as preclinical AD, progresses to mild cognitive impairment (MCI) due to AD, and eventually develops into AD dementia, following the trajectory of the so-called “AD-spectrum” (1). These AD spectrum diseases are associated with brain atrophy (2) and imaging biomarkers on MRI are important in diagnosing AD (3).

Intracranial volume (ICV), which is defined as the volume including the brain, meninges, and cerebrospinal fluid, is used to reduce the variability from different head sizes and adjust the percentiles of brain atrophy in neurodegenerative disorders (4). Several studies proposed automated brain extraction or skull stripping methods to calculate ICV by removing non-brain soft tissues including scalp, skull, and dura. These traditional methods include: Brain Surface Extractor (BSE) (5); Brain Extraction Tool (BET) (6); Brain extraction based on nonlocal Segmentation Technique (BeaST) (7); and Robust learning-based Brain Extraction system (ROBEX) (8). Recently, several studies applied deep learning techniques, particularly convolutional neural networks (CNN) (9, 10) and UNet architectures, and showed considerable performance. SynthSeg is the convolutional neural network that firstly segment brain scans of any resolutions and contrasts (11). It produces more accurate estimation of ICV, including the CSF spaces.

Currently, several MRI-based software programs for brain volume measurement have been developed for application in clinical fields (12). FreeSurfer (FS) (13) is a widely used freely available software and produces estimated ICV using the atlas scaling factor with images of an individual’s brain after transformation and registration using a 12-parameter affine transform (14). However, it requires considerable time and complex processes to analyze data and has been used mainly for research (15). NeuroQuant (NQ) (16) is a widely used software because it has a fast processing time and provides information regarding the cortices of both hemispheres and white matter volume (17). Recently, software using deep learning algorithms has been introduced with the approval of the Korean Ministry of Food and Drug Safety (K-FDA): InBrain (18, 19), DeepBrain (3, 20), and ASTROSCAN (12).

However, there are differences among several available software programs for determining volume measurements including total ICV (15, 16, 21–23). Previous studies have used brain extraction or skull-stripping techniques. However, only a few studies reported a direct segmentation method of ICV using deep learning based automatic method because automatic outlining of the exact CSF spaces dividing from adjacent structures is often complicated with using segmented images.

We aimed to develop and validate an UNet architecture based automatic segmentation method for determining the ICV using T1-weighted and T2-FLAIR MRI and to compare the accuracy of ICV segmentation with NQ, FS, and SynthSeg in patients on the AD clinical spectrum.

## Materials and methods

### Study population

The institutional review board approved this retrospective, single-institution study with a waiver of informed consent. Patients who visited Asan Medical Center from March 2017 to October 2019 were retrospectively selected from their electronic medical records. The inclusion criteria were as follows: (a) patients who visited the memory clinic and were clinically diagnosed with AD or MCI or were cognitively normal (CN) and (b) patients who underwent brain MRI with a protocol for dementia. Of 810 potentially eligible patients, 29 patients with poor image quality or other underlying pathologies causing memory impairment were excluded.

**Figure 1:**
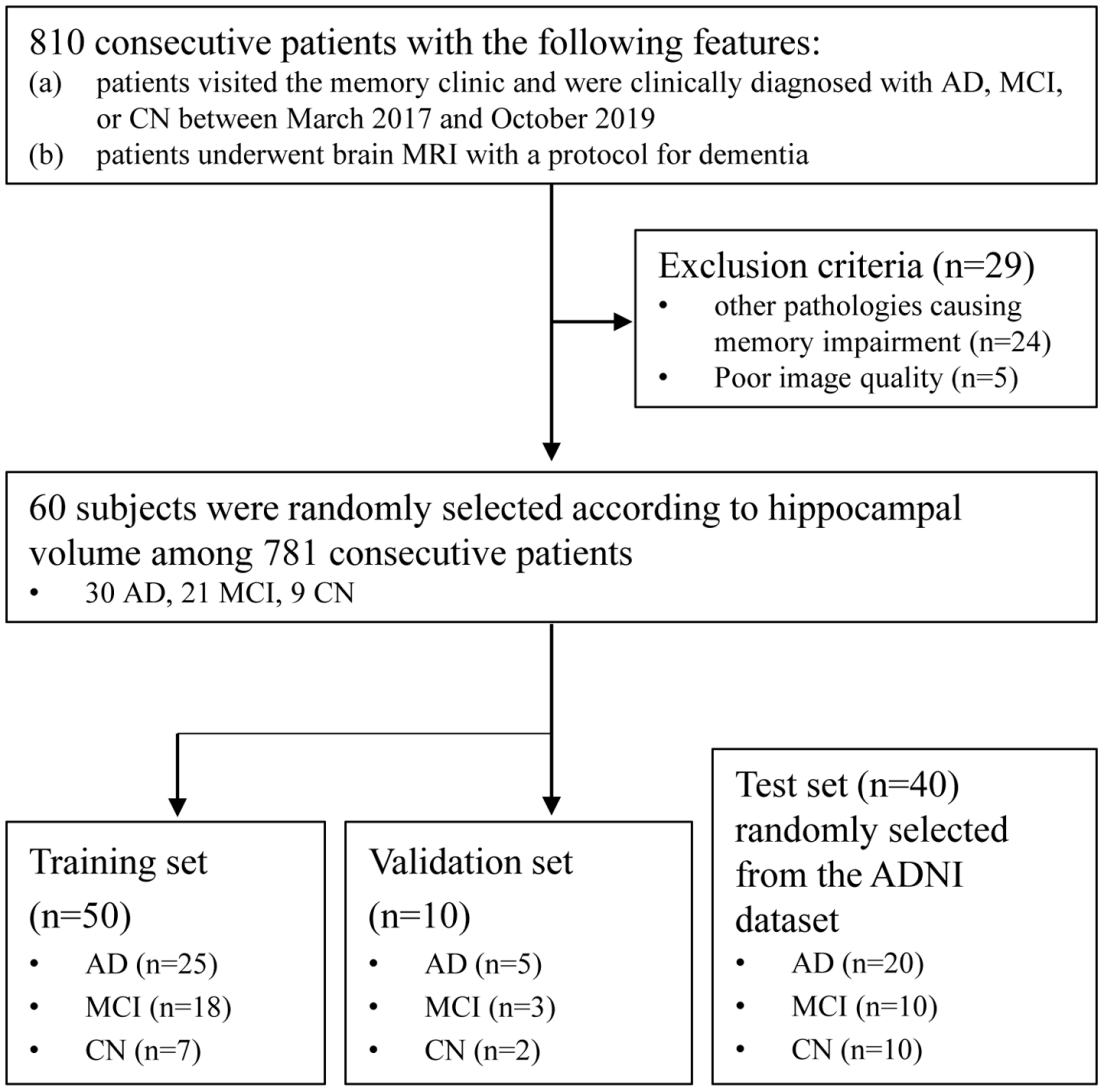
Patient flow diagram of this retrospective cohort. AD, Alzheimer’s disease; MCI, Mild cognitive impairment; CN, Cognitively normal; ADNI, Alzheimer’s Disease Neuroimaging Initiative.

Among 781 patients, 60 subjects were randomly selected according to their hippocampus volume measured by commercially available deep learning-based software (VUNO MED-DeepBrain) to ensure an even distribution of the degree of hippocampal atrophy (3). The selected subjects were split randomly into training (*n* = 50) and validation (*n* = 10) sets. For the test set, we randomly selected 40 patients from Alzheimer’s Disease Neuroimaging Initiative (ADNI) dataset (20 with AD, 10 with MCI, 10 CN) (Figure 1).

Patients with MCI and AD were diagnosed using neuropsychological evaluations based on the diagnostic guidelines of the National Institute on Aging–Alzheimer’s Association workgroups (24, 25). Patients without abnormalities on neuropsychological evaluations were classified as CN.

### Image acquisition

A routine MRI protocol was acquired using a 3.0-T system (Ingenia CX; Philips Medical Systems, Best, Netherlands) with an eight-channel head coil. All patients underwent the MRI protocol for dementia in our institution, and 3D fast field echo (FFE) T1-weighted image and two-dimensional FLAIR image were used for ICV segmentation. The parameters of images were as follows: 3D FFE T1-weighted imaging [TR/TE = 6.5/2.9; slice thickness = 1 mm; field of view (FOV) = 211 × 256 × 256 mm; flip angle 9°], FLAIR imaging (TR/TE = 9,000/125; slice thickness = 4 mm; inversion time = 2,500 ms; FOV = 220 × 220 mm).

### Deep learning-based ICV segmentation model development and volumetry

At the preprocessing stage, an original input image (3D T1-weighted MRI image) was conformed to set voxel spacing (1.0, 1.0, 1.0), image dimensions (256, 256, 256), and voxel intensity (between 0.0 and 255.0) (unscaled images). We further evaluated the effects of the voxel intensity range on the segmentation performance by setting it between 0.0 and 1.0 (scaled images). We implemented additional augmentations such as random affine transformation to enhance the model performance during the training phase. Despite the 3D structure of the brain MRI scans, we exploited only axial slices to perform segmentation in a 2.5D setting. Given a conformed (256, 256, 256) image (axes ordered by sagittal, axial, and coronal), we neglected the top and bottom 20 axial slices since there were no regions of interest. We concatenated two adjacent slices on each slice input, regarding an input slice as a three-channel image.

The proposed deep learning model exploits the basic 2D UNet architecture (26) with a Resnet34 encoder, which is a widespread neural network architecture in medical image segmentation and has achieved state-of-the-art performance for several tasks. The model comprises an encoder-decoder structure and skip connections. The encoder extracts latent features from an input image, while the decoder generates a segmentation mask from the latent feature vector. Skip connection improves high-level feature learning. Moreover, the use of a residual structure in the encoder preserves more high-level features during the feature extraction process; therefore, it leads to a significant increase in segmentation performance in multimodal settings.

**Figure 2:**
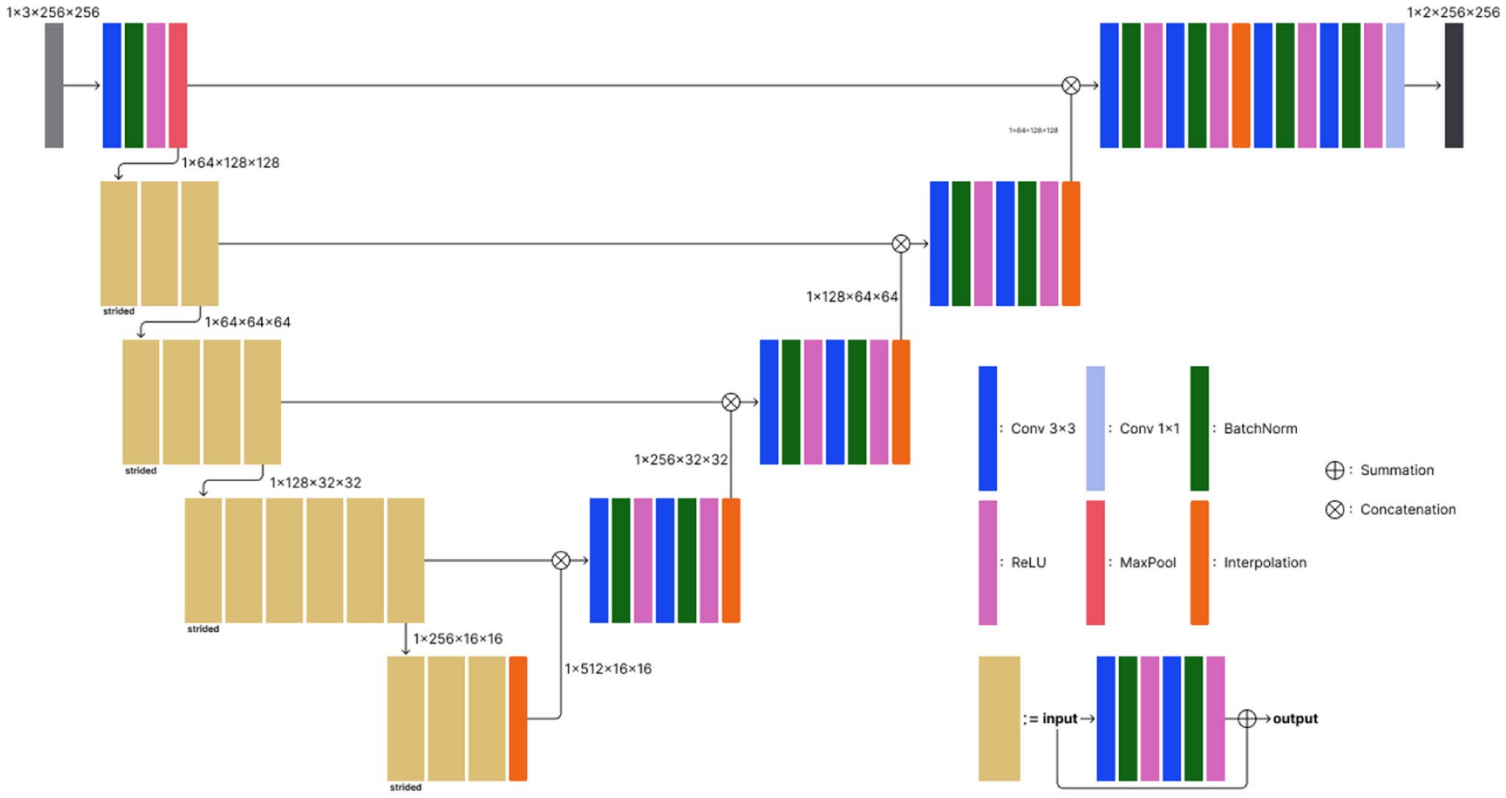
Model architecture of the proposed deep learning-based ICV segmentation model. The model exploits the basic 2D UNet architecture, which consists of five encoder and four decoder layers. Conv 3 × 3, convolutional layer with a kernel size of 3 × 3; BatchNorm, batch normalization; ReLU, rectified linear unit; MaxPool, maxpooling.

The 2D UNet model consists of five encoder and four decoder layers (Figure 2). The initial encoder layer consists of a convolutional layer with a kernel size of 3 × 3, followed by batch normalization and rectified linear unit (ReLU) activation, and a maxpooling layer to downsample the spatial features. The following encoder layers comprise multiple convolutions similar to the initial encoder layer, except for the skip connection, which facilitates more stable optimization. Furthermore, unlike the initial layer in which maxpooling comes at the end, the very first convolutions in the other encoder layers perform strided convolution to reduce the spatial dimension. Each decoder block exploits bilinear interpolation to double the spatial dimension while halving the channel dimension. Skip connection concatenates features from each encoder block and their corresponding decoder outputs. The terminal convolution squeezes the channel dimension to 2, which is the number of classes in our ICV segmentation task. The entire processing time was 5–10 s.

For the reference standard for the ICV, manual segmentation of each axial 3D T1-weighted image was performed by a board-certified diagnostic radiologist with 5 years of experience (P.S.S.) by drawing an outline of the dura. Another board-certified expert with 12 years of experience in diagnostic radiology (C.H.S.) confirmed the segmented areas.

### Multimodal ICV segmentation

We also examined if training the model with multimodal data could improve the segmentation performance. For multimodal ICV segmentation, we used both sagittal 3D T1-weighted and axial T2-FLAIR MRI images from each subject. While the preprocessing scheme for T1-weighted images was the same as for the single modality example, T2-FLAIR images were conformed into a 256 × 256 × 35 image size, where the axial axis comes at the end. The voxel size of each T2-FLAIR image was adjusted to 0.8 × 0.8 × 5.0, and the voxel intensity was normalized between 0.0 and 255.0 or between 0.0 and 1.0. We retrieved true ICV annotations from T2-FLAIR images by registering 3D T1 ICV data tensor into the T2-FLAIR space.

During the training phase, three-channel 3D T1-weighted and T2-FLAIR MRI images were randomly sampled across the subjects. In particular, unlike 3D T1-weighted images, an axial slice of a T2-FLAIR MRI was repeatedly stacked three times to generate a three-channel input. All images were cropped into a 224 × 224 size to minimize unnecessary empty backgrounds. Afterward, we implemented random geometric augmentations including image flipping and affine transforms [scale = (0.9, 1.1), translation percentage = (−0.1, 0.1), rotation angle = (−30°, 30°)]. Furthermore, we added random intensity transforms including blur, brightness contrast, gaussian noise, and shadow to reproduce noises often occur at MRI scans. All random transforms were applied with probability = 0.1, and we used the albumentation library for image augmentation (27).

### Statistical analysis

Model performance was evaluated by measuring the Dice similarity coefficient (DSC) and average volume difference. The DSC was measured for the volumetric overlap between the manually and automated segmented volume. The volumetric DSC was calculated by the intersecting volume of two masks, normalized to their mean volume. The DSC ranges from 0 to 1, indicating more overlap close to 1. The average volume difference was assessed by calculating the percentage difference between the manually segmented ICV and automated segmented volume by our proposed model, NQ, FS, and SynthSeg. ANOVA was performed to compare the measured volumes among the segmentation methods. The statistical analysis was performed using SPSS (version 21.0 for Windows; IBM Corp.), with *p* < 0.05 defined as statistically significant.

## Results

### Patient characteristics

A total of 60 subjects were randomly selected among the patients who met the inclusion criteria: 23 subjects were male (mean age ± standard deviation, 69 ± 14 years), and 37 subjects were female (mean age, 70 ± 12 years). Of these patients, 30 were clinically diagnosed with AD, 21 were diagnosed with MCI, and nine were classified as CN. The ADNI dataset included 40 subjects: 20 subjects were male (mean age, 71 ± 9 years), and 20 subjects were female (mean age, 71 ± 10 years). Of these patients, 20 were clinically diagnosed with AD, 10 were diagnosed with MCI, and 10 were classified as CN.

### Performance of automated segmentation in calculating the ICV

The performance of our trained deep learning-based software was evaluated using T1-weighted images from the test dataset from the ADNI. Using the deep learning model trained with unscaled T1-weighted images only, the DSC was 0.982 ± 0.002. Compared with the manually segmented ICV, the average volume difference was 1.67% ± 2.87%. With scaled T1-weighted images, the DSC was 0.989 ± 0.002, and the average volume difference was 0.46% ± 0.38%. Using the multimodal deep learning model trained with unscaled T1-weighted and T2-FLAIR images, the DSC was 0.988 ± 0.002, and the average volume difference was 0.47% ± 0.35%. With scaled T1-weighted and T2-FLAIR images, the DSC was 0.987 ± 0.003, and the average volume difference was 0.67% ± 0.61%. A comparison of the performance of the single-modality and multimodal deep learning models is shown in Table 1. The performance was also evaluated in each subgroup of AD, MCI, and CN using the deep learning model trained with scaled T1-weighted images and the multimodal model trained with unscaled T1-weighted and T2-FLAIR images. In the model trained with scaled T1-weighted images, the DSC in the AD, MCI, and CN subgroups were 0.990 ± 0.002, 0.988 ± 0.002, and 0.989 ± 0.001, respectively. The average volume differences in the AD, MCI, and CN subgroups were 0.41% ± 0.35, 0.50% ± 0.41, and 0.53% ± 0.41%, respectively. In the model trained with unscaled T1-weighted and T2-FLAIR images, the DSC in the AD, MCI, and CN subgroups were 0.989 ± 0.002, 0.987 ± 0.002, and 0.987 ± 0.002, respectively. The average volume differences in the AD, MCI, and CN subgroups were 0.39% ± 0.31, 0.53% ± 0.43, and 0.58% ± 0.34%, respectively.

**Table 1:** Performance of our proposed deep learning model trained with a single modality (T1-weighted images only) and multimodality (both T1-weighted and T2-FLAIR images).

	DSC (mean ± std)	Max DSC	Min DSC	Average volume difference (%)	Max volume difference (%)
Single modality (Unscaled T1)	0.982 ± 0.002	0.994	0.919	1.67 ± 2.87	15.23
Single modality (Scaled T1)	0.989 ± 0.002	0.993	0.986	0.46 ± 0.38	1.28
Multimodality (Unscaled T1+FLAIR)	0.988 ± 0.002	0.992	0.983	0.47 ± 0.35	1.24
Multimodality (Scaled T1+FLAIR)	0.987 ± 0.003	0.993	0.978	0.67 ± 0.61	2.88

DSC, Dice similarity coefficient; Max, maximum; Min, minimum; std, standard deviation.

### Comparison of NQ, FS, SynthSeg, and our proposed model

**Figure 3:**
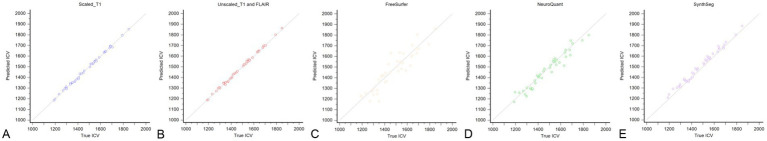
Scatterplot of the correlation between the manually segmented ICV from the T1-weighted images in the test set and automated segmented ICV determined by our proposed model trained with scaled T1-weighted images only **(A)**, unscaled both T1-weighted and T2-FLAIR images **(B)**, FreeSurfer **(C)**, NeuroQuant **(D)**, and SynthSeg **(E)**.

The performance of NQ, FS, and SynthSeg software was evaluated using T1-weighted images from the test dataset from the ADNI. There were no statistically significant differences in the measured ICVs among the measurement methods. The overall average volume difference was 2.15% ± 1.72% with NQ, 3.69% ± 2.93% with FS, and 1.88% ± 1.18% with SynthSeg. Using NQ, the average volume differences in the AD, MCI, and CN subgroups were 2.15% ± 1.54, 1.84% ± 1.77, and 2.45% ± 2.12%, respectively. Using FS, the average volume differences in the AD, MCI, and CN subgroups were 3.65% ± 2.86, 2.67% ± 2.03, and 4.78% ± 3.66%, respectively. Using SynthSeg, the average volume differences in the AD, MCI, and CN subgroups were 1.67% ± 1.01, 2.40% ± 1.17, and 1.75% ± 1.35%, respectively. A comparison of the measured volume and average volume difference in each subgroup with the three deep learning-based automatic segmentation models are shown in Table 2 and Figure 3.

**Table 2:** Comparison of the measured volume (mL) and average volume difference (%) in each subgroup for NQ, FS, SynthSeg, and our proposed deep learning model.

	AD	MCI	CN	Overall
Manual segmentation (mL)	1527.17 ± 165.76	1419.50 ± 163.47	1432.41 ± 162.79	1480.39 ± 164.51
Single modality (scaled T1) (mL)	1513.35 ± 167.78	1426.13 ± 162.20	1473.50 ± 158.52	1481.58 ± 164.04
Single modality (scaled T1) (%)	0.41 ± 0.35	0.50 ± 0.41	0.53 ± 0.41	0.46 ± 0.38
Multimodality (unscaled T1+FLAIR) (mL)	1515.51 ± 169.37	1427.93 ± 164.66	1476.11 ± 163.73	1483.76 ± 166.58
Multimodality (unscaled T1+FLAIR) (%)	0.39 ± 0.31	0.53 ± 0.43	0.58 ± 0.34	0.47 ± 0.35
NQ (mL)	1492.52 ± 163.44	1419.78 ± 156.36	1463.44 ± 174.20	1467.06 ± 163.00
NQ (%)	2.15 ± 1.54	1.84 ± 1.77	2.45 ± 2.12	2.15 ± 1.72
FS (mL)	1520.81 ± 156.69	1401.55 ± 175.42	1453.35 ± 203.61	1474.13 ± 176.66
FS (%)	3.65 ± 2.86	2.67 ± 2.03	4.78 ± 3.66	3.69 ± 2.93
SynthSeg (mL)	1534.56 ± 162.74	1456.51 ± 158.57	1498.41 ± 147.77	1506.01 ± 161.32
SynthSeg (%)	1.67 ± 1.01	2.40 ± 1.17	1.75 ± 1.35	1.88 ± 1.18

AD, Alzheimer’s disease; MCI, Mild cognitive impairment; CN, Cognitively normal.

## Discussion

In this study, we developed and validated a deep learning-based automatic ICV segmentation model using axial 3D T1-weighted and T2-FLAIR MR images, which used not brain extraction or skull stripping techniques but direct segmentation with short processing time. Our model showed excellent performance in the measurement of the ICV in every subgroup of the AD clinical spectrum. There were differences in the measured ICV among the ICV segmentation software programs, and our model outperformed the others. Therefore, our deep learning-based automatic ICV segmentation model might be considered for the accurate evaluation of brain atrophy in neurodegenerative disorders.

Numerous segmentation models have been developed and they have enhanced the performance of ICV segmentation. In this study, we compared the average volume differences with clinically available ICV segmentation software including FS, NQ, and SynthSeg. For this comparison, we selected our deep learning model trained with scaled T1-weighted images and unscaled T1-weighted and T2-FLAIR images, which showed better performance with the single-modality and multimodality models. All of the automated segmentation models and software programs showed no significant differences compared with the manually segmented ICV, suggesting good performance. The overall average volume difference in our model showed minimal differences with the manually segmented ICV (0.46% ± 0.38% in the single-modality model and 0.47% ± 0.35% in the multimodality model), demonstrating better accuracy than FS (3.69% ± 2.93%), NQ (2.15% ± 1.72%), and SynthSeg (1.88% ± 1.18%). Previous studies have compared NQ and FS and showed a high correlation (16, 17, 21, 28). The segmentation method of NQ is similar to that of FS, but it utilizes a different atlas, an independent code base, and separate methods for normalization of intensity and correction of gradient distortion to accommodate for scanner-specific acquisition-level differences (16). In contrast, our proposed segmentation model used an atlas-free deep learning model. With the addition of random augmentations, our deep learning model learned preprocessing and protocol-invariant features for ICV segmentation from training images. On the other hand, atlas-based models are inevitably sensitive to imaging protocols. Hence, the proposed model was less prone to overfitting than the other methods and thus demonstrated enhanced test accuracy. Another advantage of our model was the short processing time (5–10 s) compared with FS (7 h) and NQ (10 min) (16, 29). This advantage is essential for application in actual clinical fields.

We also compared the average volume difference in the AD, MCI, and CN subgroups. Similar to the overall average volume difference, our model showed better accuracy than FS, NQ, and SynthSeg in each subgroup. In addition, both the single-modality and multimodality models showed a lower volume difference in the AD subgroup than in the MCI and CN subgroups. Our model used an atlas-free deep learning model, and this might have led to good performance despite atrophic changes in the brain parenchyma. As the ICV is used to adjust the degree of brain atrophy in patients with neurodegenerative disorders and not in normal patients, this can be another benefit for clinical application.

The DSC was calculated to provide a quantitative assessment of the performance of our segmentation model. The overall DSC of our model was 0.989 ± 0.002 in the single-modality segmentation model trained with scaled T1-weighted images only and 0.988 ± 0.002 in the multimodal segmentation model trained with both unscaled T1-weighted and T2-FLAIR images. As the DSC represents spatial overlap and reproducibility (30), our model demonstrated near complete spatial overlap and good reproducibility.

**Figure 4:**
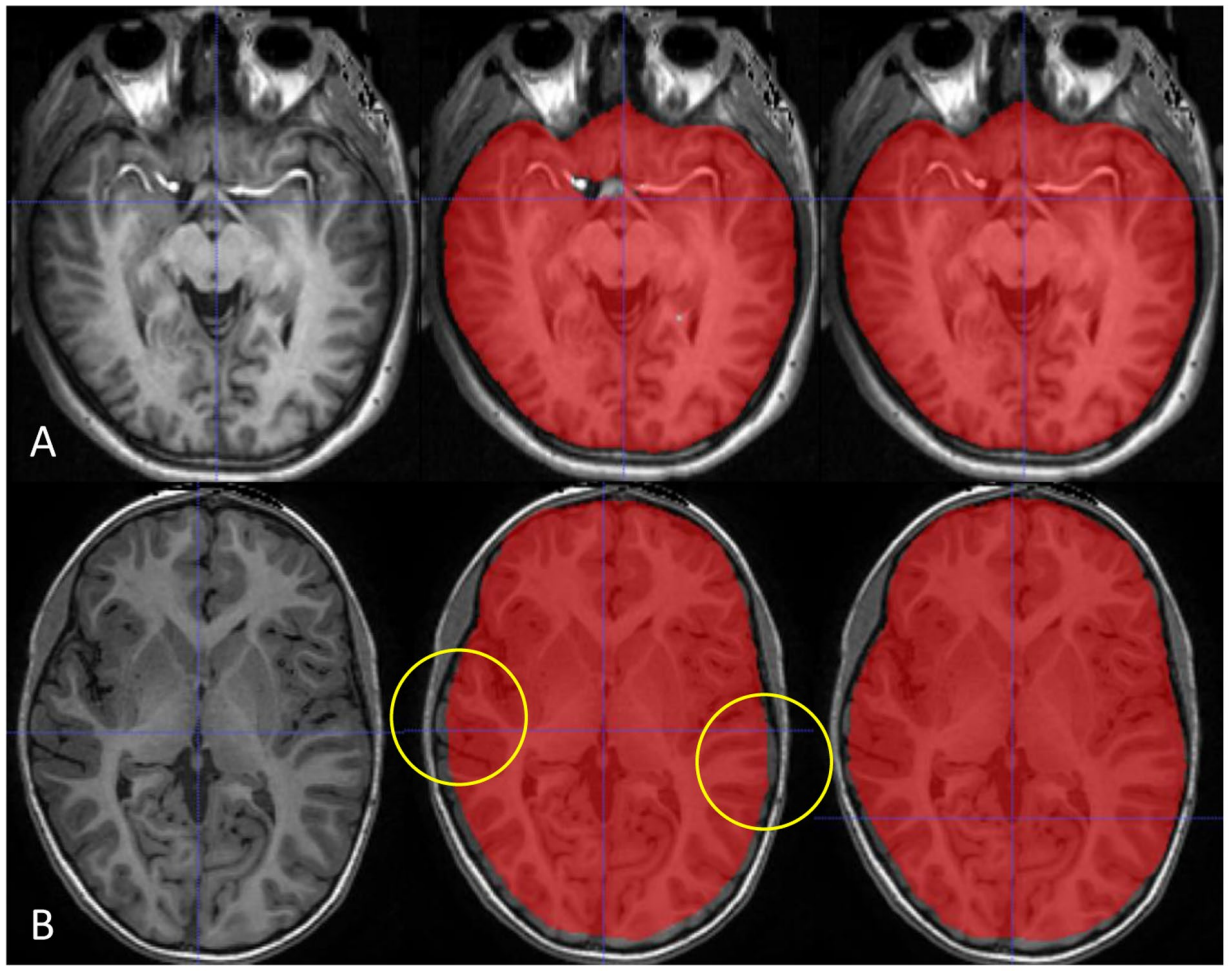
Our multimodality deep learning model shows robustness in various protocols and patient ages. The multimodal model (right) shows advantages in enhanced T1-weighted images around hyperintense enhanced vessels **(A)** and images from a young patient without atrophy images **(B)** compared with the single-modality model (middle).

As deep learning algorithms advance, numerous algorithms for ICV segmentation have been developed. Ntiri et al. (22) compared the DSC of their segmentation model with those of other ICV extraction models and found values of 0.976 ± 0.016 and 0.960 ± 0.027 of iCVMapper and FS, respectively, using T1-weighted images. In addition, the DSC increased when using a multi-contrast network using T1-weighted, T2-weighted, and FLAIR sequences as inputs. In our study, the single-modality deep learning model trained with scaled T1-weighted images showed the best performance as the scaling of input data can achieve improvement in the training process. However, the performance of our multimodal deep learning model was not inferior to that of the single-modality model. In addition, although not included in the results of our study, the multimodal model showed robustness in various protocols and patient ages (Figure 4). Therefore, we expect the advantage of the multimodal model for clinical application, and further studies are needed.

Developing individual models for a single image modality is inefficient in clinical practice since initializing multiple models with limited hardware causes memory burden. Hence, a multimodal segmentation scheme is desired when a single task is performed on several types of images (sagittal 3D T1-weighted and axial T2-FLAIR images in our case). HyperDenseNet (31), for example, is a multimodal segmentation network for T1- and T2-weighted images that is designed for brain tissue segmentation. However, without sophisticated manipulations of the network architecture, we found that using the same architecture with the single-modality segmentation model was suitable enough for the multimodal ICV segmentation task.

There were several limitations in this study. First, although we randomly selected a small number of subjects from a single institution, there was still potential for selection bias. Second, we did not consider reproducibility with different MRI scanners or protocols. Several factors including MRI parameters, magnetic field strength, and scanner models can influence the results of volumetry. Particularly, FLAIR imaging used in our model can be appeared variable based on the acquisition parameters. Third, we did not test other institutional data or perform a “real-world” external test. All training data used in our model originated from a single protocol from a single MRI scanner. Therefore, this is essential for application in an actual clinical setting. Further studies are warranted for validation.

## Conclusion

Our deep learning-based automatic ICV segmentation model showed excellent performance in the automatic evaluation of the ICV. Our model might be considered for the accurate evaluation of brain atrophy in neurodegenerative disorders.

## Data availability statement

The original contributions presented in the study are included in the article/supplementary material, further inquiries can be directed to the corresponding author.

## Ethics statement

The studies involving humans were approved by the Asan Medical Center Institutional Review Board. The studies were conducted in accordance with the local legislation and institutional requirements. Written informed consent for participation was not required from the participants or the participants’ legal guardians/next of kin in accordance with the national legislation and institutional requirements.

## Author contributions

All authors listed have made a substantial, direct, and intellectual contribution to the work and approved it for publication.

## Funding

This work was supported by the National Research Foundation of Korea (NRF-2021R1C1C1014413) and National Research Foundation of Korea funded by the Ministry of Education (NRF-2022R1I1A1A01072397).

## Conflict of interest

The authors declare that the research was conducted in the absence of any commercial or financial relationships that could be construed as a potential conflict of interest.

## Publisher’s note

All claims expressed in this article are solely those of the authors and do not necessarily represent those of their affiliated organizations, or those of the publisher, the editors and the reviewers. Any product that may be evaluated in this article, or claim that may be made by its manufacturer, is not guaranteed or endorsed by the publisher.

## Abbreviations

AD: Alzheimer’s disease
MCI: mild cognitive impairment
CN: cognitively normal
FS: FreeSurfer
NQ: NeuroQuant
ICV: intracranial volume
DSC: Dice similarity coefficient
