# Corrigendum: Development and validation of a deep learning-based automatic segmentation model for assessing intracranial volume: comparison with NeuroQuant, FreeSurfer, and SynthSeg

**DOI:** 10.3389/fneur.2023.1334962

**Published:** 2023-11-29

**Authors:** Pae Sun Suh, Wooseok Jung, Chong Hyun Suh, Jinyoung Kim, Jio Oh, Hwon Heo, Woo Hyun Shim, Jae-Sung Lim, Jae-Hong Lee, Ho Sung Kim, Sang Joon Kim

**Affiliations:** ^1^Department of Radiology and Research Institute of Radiology, Asan Medical Center, University of Ulsan College of Medicine, Seoul, Republic of Korea; ^2^R&D Center, VUNO, Seoul, Republic of Korea; ^3^Department of Neurology, Asan Medical Center, University of Ulsan College of Medicine, Seoul, Republic of Korea

**Keywords:** deep learning, artificial intelligence, brain, intracranial volume segmentation, neurodegenerative disease

In the published article, there was an error in affiliation 1 and 3. Instead of “^1^Department of Radiology, Asan Medical Center, Seoul, Republic of Korea; ^3^Department of Neurology, Asan Medical Center, College of Medicine, University of Ulsan, Ulsan, Republic of Korea”, it should be “^1^Department of Radiology and Research Institute of Radiology, Asan Medical Center, University of Ulsan College of Medicine, Seoul, Republic of Korea; ^3^Department of Neurology, Asan Medical Center, University of Ulsan College of Medicine, Seoul, Republic of Korea”.

The authors apologize for this error and state that this does not change the scientific conclusions of the article in any way. The original article has been updated.

